# Draft Genome Sequence of Pasteurella multocida Serotype A Strain MOR19, Isolated in Morocco

**DOI:** 10.1128/MRA.00867-21

**Published:** 2021-10-14

**Authors:** Noha Semmate, Zineb Boumart, Zahra Bamouh, Slimane Khayi, Khalid Omari Tadlaoui, Siham Fellahi, Mehdi El Harrak

**Affiliations:** a Research and Development Department, Multi-Chemical Industry, Mohammedia, Morocco; b Institute of Agronomy and Veterinary Medicine Hassan II, Rabat, Morocco; c Biotechnology Research Unit, CRRA-Rabat, National Institute for Agricultural Research, Rabat, Morocco; University of Maryland School of Medicine

## Abstract

Pasteurella multocida causes pneumonia in large ruminants. In this study, we determined the genome sequence of the capsular serotype A Pasteurella multocida strain MOR19, isolated from a calf that died from acute pneumonia.

## ANNOUNCEMENT

Pasteurella multocida is a widespread Gram-negative, nonmotile, coccobacillary, and nonsporing opportunistic pathogen. Strains of Pasteurella multocida can be classified into five capsular serogroups (A, B, D, E, and F) (1). The importance of Pasteurella multocida is well established as an etiologic factor of hemorrhagic septicemia and pneumonia in cattle and sheep, which is a major cause of economic loss and mortality. The disease complex seems to be caused by a combination of respiratory viruses, bacterial infections, stress, and other environmental factors (2).

Recently, Pasteurella multocida strain MOR19 was isolated from a calf that died from acute and severe pneumonia in the north region of Morocco (Casablanca Province). Lung tissue fragments were used to inoculate tryptic soya agar plates supplemented with 5% sheep blood. The plates were incubated at 37°C for 24 h under aerobic conditions, and the isolated colonies revealed Gram-negative coccobacilli that were submitted for identification using biochemical tests (catalase, oxidase, and indole) and using an API 20 NE kit (bioMérieux). The isolate was identified as Pasteurella multocida.

A multiplex PCR assay was conducted for molecular characterization of the capsular antigens of Pasteurella multocida using specific primer sets for the serogroups A, B, D, E, and F, according to the method of Townsend et al. (3). The serogroup was identified according to the following two criteria: (i) primer sets located within the unique gene for each of the five serogroups (hyaD, bcbD, dcbF, ecbJ, and fcbD) and (ii) an amplicon length sufficient to allow clear size discrimination. The multiplex PCR mixture contained the six primer sets, at a concentration of 3.2 μM 1× standard *Taq* (Mg free), 2 mM MgCl_2_, each deoxynucleoside triphosphate at a concentration of 0.2 Mm, and 0.5 U of *Taq* DNA polymerase. The PCR cycling conditions consisted of an initial denaturation at 95°C for 5 min, followed by 35 cycles of denaturation at 95°C for 30 s, annealing at 55°C for 30 s, extension at 72°C for 60 s, and a final elongation at 72°C for 5 min. The amplified products were separated by electrophoresis in 1% agarose gel and visualized by ethidium bromide staining. Amplification reactions were performed using Gene Amp 9700 (Applied Biosystems). The isolate was identified as Pasteurella multocida serotype A, strain MOR19.

To understand the mechanism of pasteurellosis in ruminants, we proceeded to full genome sequencing of the isolate. Genomic DNA was extracted from fresh bacterial culture, using the Isolate II genomic DNA kit (Bioline), and resuspended in 100 μl of nuclease-free distilled water. After DNA fragmentation, 500-bp genomic DNA fragments were selected using E-Gel SizeSelect agarose gels. Library construction was performed using the NEBNext Ultra II directional DNA library prep kit for Illumina with TruSeq adapter sequences. The sequencing was performed on the Illumina NovaSeq 6000 platform using 2 × 150-bp sequence read mode.

The raw data were trimmed to remove low-quality reads (cutoff, 0.05) and ambiguous nucleotides (*n* ≤ 2) using CLC Genomics Workbench v12. In total, 12,471,806 reads were recovered. *De novo* assembly was carried out using CLC Genomics Workbench v12 with default parameters (length fraction, 0.5; similarity, 0.8), generating 42 contigs (>2,000 bp) with an average coverage of 800×. The largest contig was 425,061 bp, and the smallest was 3,021 bp. The *N*_50_ parameter for the contigs was estimated to be 82,614 bp.

The assembled Pasteurella multocida strain MOR19 genome sequence is 2,315,687 bp, with a G+C content of 40%. Gene prediction using the NCBI Prokaryotic Genome Annotation Pipeline (PGAP) (4) resulted in 2,213 genes, including 2,165 coding DNA sequences (CDS), 43 tRNAs, and 5 rRNAs.

### Data availability.

This whole-genome shotgun project has been deposited at DDBJ/ENA/GenBank under accession number JADPRV00000000. The version described in this paper is version JADPRV000000000.1. The Illumina reads are available in the SRA under accession number SRR13089847.
